# Cancer-Associated Fibroblasts Regulate Kinase Activity in Mesothelioma Cell Lines via Paracrine Signaling and Thereby Dictate Cell Faith and Behavior

**DOI:** 10.3390/ijms23063278

**Published:** 2022-03-18

**Authors:** Alexander Mathilakathu, Michael Wessolly, Elena Mairinger, Hendrik Uebner, Daniel Kreidt, Luka Brcic, Julia Steinborn, Kristina Greimelmaier, Jeremias Wohlschlaeger, Kurt Werner Schmid, Fabian D. Mairinger, Sabrina Borchert

**Affiliations:** 1 Institute of Pathology, University Hospital Essen, University of Duisburg Essen, 45147 Essen, Germany; alexander.mathew98@yahoo.de (A.M.); michael.wessolly@uk-essen.de (M.W.); elena.mairinger@gmail.com (E.M.); daniel.kreidt@stud.uni-due.de (D.K.); julia.steinborn@uk-essen.de (J.S.); KW.Schmid@uk-essen.de (K.W.S.); fabian.mairinger@uk-essen.de (F.D.M.); 2 Department of Pulmonary Medicine, University Hospital Essen—Ruhrlandklinik, University of Duisburg Essen, 45147 Essen, Germany; Hendrik.beckert@rlk.uk-essen.de; 3 Diagnostic and Research Institute of Pathology, Medical University of Graz, 8036 Graz, Austria; luka.brcic@medunigraz.at; 4 Department of Pathology, Diakonissenkrankenhaus Flensburg, 24939 Flensburg, Germany; greimelmaierkr@diako.de (K.G.); jeremias.wohlschlaeger@uk-essen.de (J.W.)

**Keywords:** pleural mesothelioma, cancer-associated fibroblasts, kinases, phosphorylation, microenvironment, MAPK signaling, MEK and ERK inhibitors

## Abstract

Background: Malignant pleural mesothelioma (MPM) has an infaust prognosis due to resistance to systemic treatment with platin-analoga. MPM cells modulate the immune response to their benefit. They release proinflammatory cytokines, such as TGF-ß, awakening resting fibrocytes that switch their phenotype into activated fibroblasts. Signaling interactions between cancer cells and cancer-associated fibroblasts (CAFs) play an integral part in tumor progression. This study aimed to investigate the role CAFs play in MPM progression, analyzing the impact this complex, symbiotic interaction has on kinase-related cell signaling in vitro. Methods: We simulated paracrine signaling in vitro by treating MPM cell lines with conditioned medium (CM) from fibroblasts (FB) and vice versa. NCI-H2052, MSTO-211H, and NCI-H2452 cell lines representing the three mayor MPM subtypes, while embryonal myofibroblast cell lines, IMR-90 and MRC-5, provide a CAFs-like phenotype. Subsequently, differences in proliferation rates, migratory behavior, apoptosis, necrosis, and viability were used as covariates for data analysis. Kinase activity of treated samples and corresponding controls were then analyzed using the PamStation12 platform (PamGene); Results: Treatment with myofibroblast-derived CM revealed significant changes in phosphorylation patterns in MPM cell lines. The observed effect differs strongly between the analyzed MPM cell lines and depends on the origin of CM. Overall, a much stronger effect was observed using CM derived from IMR-90 than MRC-5. The phosphorylation changes mainly affected the MAPK signaling pathway.; Conclusions: The factors secreted by myofibroblasts in fibroblasts CM significantly influence the phosphorylation of kinases, mainly affecting the MAPK signaling cascade in tested MPM cell lines. Our in vitro results indicate promising therapeutic effects by the use of MEK or ERK inhibitors and might have synergistic effects in combination with cisplatin-based treatment, improving clinical outcomes for MPM patients.

## 1. Introduction

Malignant pleural mesothelioma (MPM) is a malignancy originating from the pleural mesothelium, a serosal membrane covering the thoracic cavity. This rare type of cancer is heavily associated with asbestos exposure and has an infaust prognosis with median survival ranging from 9–15 months after initial diagnosis [1]. Multimodality treatment consisting of chemotherapy, surgery, and/or radiotherapy is centered around surgical resection in the early stages. Systemic chemotherapy options include antifolate pemetrexed in combination with cisplatin [2] or carboplatin [3,4,5,6]. Treatment with cisplatin results in a response rate of merely 14% [7], while response rates with carboplatin range from 6% to 16% [7,8]. Recent studies report extended median overall survival rates of 14.1 months to 18.1% by treatment with ipilimumab and nivolumab (NCT02899299) [9].

Most human mesothelial cells affected by asbestos-related microfibers undergo apoptosis. Some escape the programmed cell death and accumulate pro-oncogenic mutations, resulting in malignant transformation and MPM development [10]. These malignant cells modulate the immune response to their benefit, releasing immunomodulatory cytokines, such as TGF-ß, awakening resting fibrocytes that switch their phenotype into activated fibroblasts. In turn, these cancer-associated fibroblasts (CAFs) change the local fiber structure in a process known as desmoplasia [11,12]. Furthermore, signaling interactions between cancer cells and CAFs play an integral part in tumor progression [13]. At the same time, advanced desmoplasia and stromal changes have been identified as factors for a poor prognosis in MPM patients [14]. In addition, over the past decade, understanding of the underlying effect has significantly increased by identifying major components of the apoptotic program and the processes regulating their activation. Kinases that have been suggested to play a role in apoptosis are the mitogen-activated protein kinase (MAPK) family, specifically p42/44 ERK, p38 MAPK and c-Jun N-terminal kinase (JNK), cyclic AMP-dependent protein kinase A (PKA), protein kinase B (PKB, also known as AKT), and protein kinase C (PKC) [15].

New therapeutic approaches are needed to overcome the high mortality of MPM, bearing in mind that the incidence peak of MPM is expected within this decade [16]. Therefore, we aimed to analyze the role CAFs have in MPM progression by analyzing the impact this complex, symbiotic interaction has on kinase-related cell signaling in vitro, hopefully identifying new potential therapeutic targets.

This section may be divided by subheadings. It should provide a concise and precise description of the experimental results, their interpretation, as well as the experimental conclusions that can be drawn.

### 1.1. The Treatment Effect with FB Derived CM on Overall Kinase Pattern in MPM

**Figure 1:**
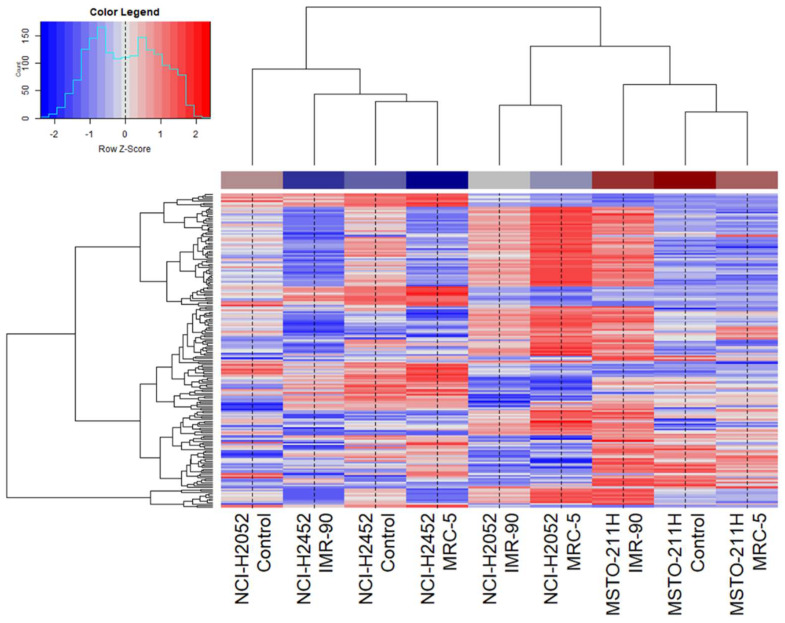
Unsupervised clustering of treated cell lines and controls. MPM cell lines (NCI−H2052, NCI−H2452, and MSTO−211H) were treated with conditioned medium of fibroblast cell lines IMR-90 or MRC-5. In controls, conditioned medium of the respective MPM cell line itself was used.

In search of significant changes in phosphorylation pattern after treatment with CM, GSK3B, and MK12 revealed a significant upregulation of two- to threefold from baseline (GSK3B: *p* = 0.036, MK12: *p* = 0.044) (Figure 1).

The observed effect differs strongly between the analyzed MPM cell lines and depends on the origin of CM. Overall, a much stronger effect could be observed using CM conditioned by IMR-90 than those conditioned by MRC-5, the two activated myofibroblast cell lines used for conditioning of the medium by secretion of (soluble) factors. In general, epithelioid NCI-H2052 cells treated with CM of FB showed a distinct phosphorylation pattern illustrated by unsupervised clustering, with many hyperphosphorylated phosphosites (blue shaded) (Figure 1). Biphasic MSTO-211H cells showed minor differences in phosphorylation patterns after treatment with CM of MRC-5 compared to control. However, phosphorylation increases dramatically after treatment with CM derived of IMR-90 cells, leading to a similar phosphorylation pattern observed in NCI-H2052 cells after treatment with FB CM. Comparing the patterns of the treated NCI-H2452 sarcomatoid cells with those of the control cells, a significant reduction in phosphorylation of most of the phosphosites analyzed can be seen (shaded red). This observed effect was significantly more pronounced after treatment with IMR-90 derived CM. To conclude, all studied MPM cell lines treated with CM derived from myofibroblast cells—especially when looking at IMR-90 cells—showed a wide-ranging altered phosphorylation pattern compared to controls. This results in activation of kinase activity in NCI-H2052 and MSTO-211H cells and their inhibition in NCI-H2452 cells.

### 1.2. Viability, Apoptosis, and Invasiveness of MPM Cells Treated with FB Derived CM First Bullet

The viability of treated cell lines was analyzed using enzyme activity-based assays. Overall, enhanced phosphorylation of RON, MAPK3, CD3Z, EGFR, GSK3B, and JAK1 showed significant associations with increased viability rates in each treated cell line, compared to cell lines treated with control medium (Supplementary Table S1).

**Figure 2:**
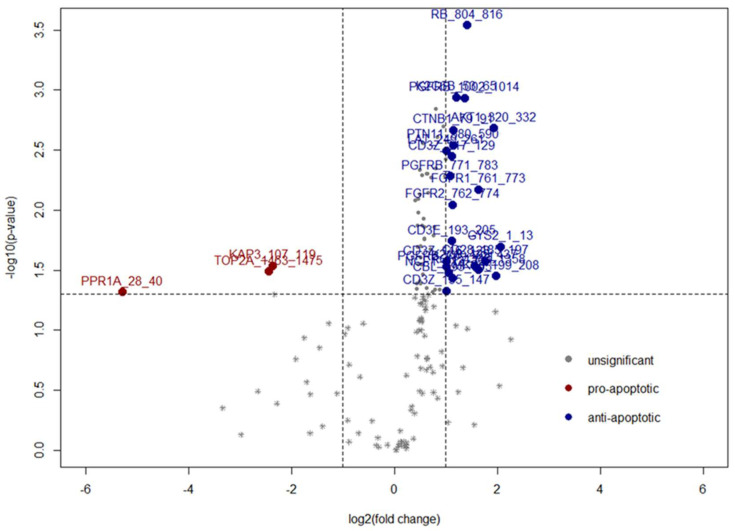
Volcano plot of phosphotsites associated with changes in apoptosis of FB CM-treated MPM cell lines.

Moreover, enhanced apoptotic rates are associated with the phosphorylation of multiple phosphosites (Figure 2, linear regression model). Altogether, 39 phosphosites were associated with significant elevation of apoptotic rates. Increased phosphorylation of 36 phosphosites in 32 protein targets including RB, ARAF, AKT1, FAK2, etc. (Supplementary Table S2, showing positive Rho, spearman test) was associated with elevated apoptotic rates (Figure 2). Contrarily, phosphorylation of EPB42, STK6, and CREB was decreased and thus associated with increased apoptosis (Supplementary Table S2, showing negative Rho, spearman test).

In addition, the invasive behavior of MPM cells under the influence of FB CM was assessed by the implementation of cell migration and invasion assays for each MPM cell line. Kinase activity analysis of these treated cells showed a significant association between decreased phosphorylation of CDK4 (*p* = 0.034), resulting in increased migration behavior of MPM cells treated with FB CM.

### 1.3. Gene Set Enrichment Analysis

To identify involved pathways and biological functions/categories beyond phosphorylation patterns after MPM cell lines treatment, a gene set enrichment analysis (GSEA) was performed. GSEA utilizes molecular interaction networks outlined by the Kyoto Encyclopedia of Gene and Genomes (KEGG) to map out increased target phosphorylation in a specific molecular pathway, depending on a response variable.

**Figure 3:**
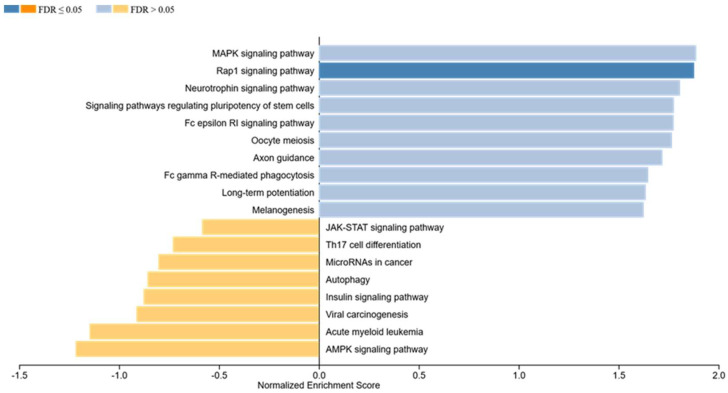
Gene set enrichment analysis in MPM cells being treated with FB CM. Among others, particularly the MAPK-RAP1 and the Fc receptor signalling pathway showed significant associations. These pathways were activated in treated MPM cell lines.

GSEA revealed highly significant associations with the Rap1 signaling pathway (*p* ≤ 0.001, normalized enrichment score: 1.88), Oocyte meiosis (*p* ≤ 0.001, normalized enrichment score: 1.77), the Fc epsilon RI signaling pathway (*p* ≤ 0.001, normalized enrichment score: 1.78), the MAPK signaling pathway (*p* = 0.003, normalized enrichment score: 1.89), the Neurotrophin signaling pathway (*p* ≤ 0.001, normalized enrichment score: 1.81), the signaling pathways regulating pluripotency of stem cells (*p* = 0.002, normalized enrichment score: 1.78), Long-term potentiation (*p* = 0.009, normalized enrichment score: 1.64) and Axon guidance (*p* = 0.005, normalized enrichment score: 1.72). Furthermore, significant enrichment has been calculated for the Fc gamma R-mediated phagocytosis (*p* = 0.013, normalized enrichment score: 1.65) and Melanogenesis (*p* = 0.032, normalized enrichment score: 1.63). The mentioned pathways all show activation in MPM cell lines treated with FB CM (Figure 3).

Details of the GSEA regarding FB CM treatment, including normalized enrichment score, the *p*-value of enrichment, exact targets included in the gene sets, and those differentially regulated, can be found in Supplementary Table S3.

Regarding cell-state effects in the context of specific phosphorylation patterns of phosphosites, a set of signaling pathways similar to those enriched due to FB CM has been found in the GSEA analysis.

**Figure 4:**
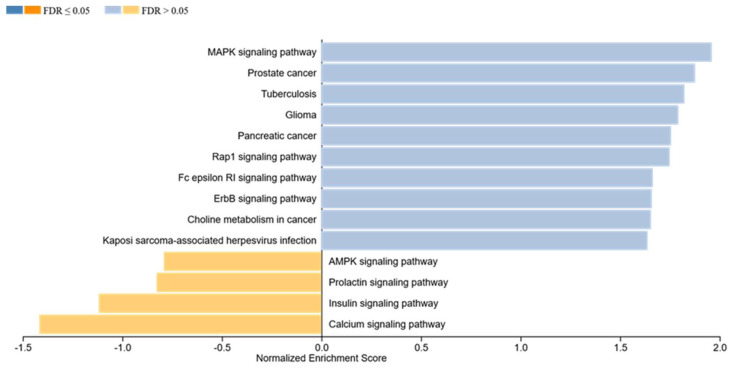
Gene set enrichment analysis in relation to apoptosis in MPM cells being treated with FB CM. Particularly, the MAPK-RAP1 and the Fc receptor signaling pathway showed significant associations. These pathways were activated in treated MPM cell lines.

**Figure 5:**
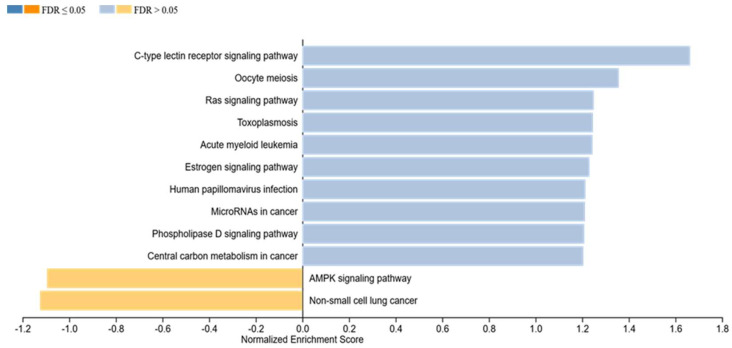
Gene set enrichment analysis in relation to viability of MPM cells being treated with FB CM. Particularly, the C-type lectin receptor signaling pathway showed significant associations (*p* = 0.01). This pathway was activated in treated MPM cell lines.

Concerning induction of apoptosis, our calculations result in highly significant enrichment of the MAPK signaling pathway (*p* = 0.003, normalized enrichment score: 1.96), Prostate cancer (*p* ≤ 0.001, normalized enrichment score: 1.88), Glioma (*p* = 0.009, normalized enrichment score: 1.79), and Tuberculosis (*p* = 0.004, normalized enrichment score: 1.82) (Figure 4). Furthermore, significant enrichment has been calculated for the Rap1 signaling pathway (*p* = 0.016, normalized enrichment score: 1.75), Pancreatic cancer (*p* = 0.020, normalized enrichment score: 1.76), Choline metabolism in cancer (*p* = 0.037, normalized enrichment score: 1.65), Kaposi-sarcoma-associated herpesvirus infection (*p* = 0.032, normalized enrichment score: 1.64), ErbB signaling pathway (*p* = 0.023, normalized enrichment score: 1.66), and Fc epsilon RI signaling pathway (*p* = 0.018, normalized enrichment score: 1.66). Activation of all of those pathways is significantly associated with increased levels of apoptosis (Figure 4). Moreover, the C-type lectin receptor signaling pathway (*p* = 0.005, normalized enrichment score: 1.66) was enriched in association to elevated cell viability (Figure 5).

Details of the GSEA regarding cell state, including normalized enrichment score, the *p*-value of enrichment, exact targets included in the gene sets, and those differentially regulated, can be found in Supplementary Table S4 (apoptosis) and Supplementary Table S5 (viability).

## 2. Discussion

Until recently, the tumor microenvironment (TME) role and importance have not been in the focus of mesothelioma research. However, the complex interactions of extracellular signaling within TME itself and its influence on tumor cells gain more attention, especially regarding new potential treatment options. Interactions between CAFs, tumor cells, and the host’s immune system are of particular interest as they hinder an effective immune response and aid tumor progression, thus ultimately leading to failure of chemotherapy [17]. Regarding one of the roles of CAFs, Okuyama, et al. reported that the interesting propose of novel mechanism of anaplastic transition (APT) on tongue cancer, in which epithelial cancer cells concurrently develop mesenchymal features, which is achieved by pathways other than EMT. Concurrent expression of cytokeratin and vimentin is not defined as EMT or partial EMT. Therefore, APT is characterized such that epithelial cancer cells differentiate into more primitive states, which is different from EMT or partial EMT, and it may be associated with local recurrence of tongue cancer [18].

The tumor-stromal tissue comprises fibroblasts, endothelial/immune cells, and extracellular matrix (ECM) components, all different from their dormant counterparts in homeostatic tissues [19]. In homeostasis, epithelial cells are segregated from fibroblasts [20]. Resting fibroblasts have minimal transcriptional and metabolic activity; nevertheless, they play an important role in the differentiation status of adjacent epithelia through the secretion of specific signaling factors, including Wnt and bone morphogenic proteins (collagens, fibronectin, and elastin) [21,22]. Besides chemical and signaling triggers, fibroblasts in carcinogenic tissue can be activated through tumor growth (mechanotransduction) [19,23,24]. Expressing, among others, α-SMA, desmin, and fibroblast activation protein (FAP), they transform into myofibroblasts/CAFs [19].

In this study, we simulated paracrine signaling in vitro by treating MPM cell lines with conditioned medium (CM) from fibroblasts (FB) and vice versa. Limits of this study are the miss of nontumoral cells and the use of already FAP-expressing myofibroblasts. This has to be considered by analyzing the gained results. The use of a nontumoral cell line as control is challenging. The cell line MET-5a is often used as a benign version of MPM cells. However, this cell line is SV40-immortalized and thus do show altered culture performance [25]. The mesothelial cell line LP-9 could be an alternative option for use as a benign control. However, this cell line was telomerase-immortalized and must be investigated and validated [26].

We observed that the effect of treatment with FB derived CM in MPM varies for each cell line related to the overall kinase pattern. A much stronger effect was observed after the usage of CM from IMR-90 in comparison to those from MRC-5. In a previous study, we were able to identify that cell–cell interaction via cytokines seems to be of greater importance in patients with an epithelioid subtype of MPM [27]. These results are consistent with our in vitro observation in this study since most of significant changes in phosphorylation pattern also occurred in the epithelioid NCI-H2052 cell line. Paracrine signaling by CAFs generally consists of interleukins, cytokines, and growth factors such as hepatocyte growth factor (HGF), C-X-C chemokine receptor type 4 (CXCR4), and interleukin 6 [17,28]. Their release also activates, among other things, pathways, which were upregulated in our approach, as the most substantial phosphorylation pattern changes were seen in the MAPK and RAF signaling pathways. Tumor cells benefit from the activation of these pathways, as they affect tumor growth, metastatic potential, and response to therapy [19]. Wen et al. observed that CAF-derived IL-32 is specifically bound to integrin β3 through the RGD motif, activating intracellular downstream p38 MAPK signaling in breast cancer cells [29]. This signaling increased the expression of EMT markers (fibronectin, N-cadherin, and vimentin) and promoted tumor cell invasion [29].

The MAPK pathway is a complex, interconnected signaling cascade with frequent involvement in oncogenesis, tumor progression, and drug resistance [30]. These hierarchically organized enzymes potentiate signals for cell proliferation, growth, and survival processes [30,31,32]. This signaling cascade is physiologically initiated by releasing extracellular stimuli in the form of growth factors binding and activating receptor tyrosine kinases (RTKs) covering the cell membrane. Through selective downstream activation of RAS, RAF, and MEK, malignancies frequently bypass this barrier, ultimately leading to the upregulation of the ERK1/2 transcription factor activator [30].

The results of our approach are consistent with this line of thought, as both MAPK and RAF signaling pathways are affected in FB CM-treated cell lines, especially in the NCI-H2052 cell line. Several of these interlocking kinases showed altered activation levels, probably significantly contributing to measured changes in viability and increased proliferation rates.

Concerning the induction of apoptosis, MAPK signaling pathway showed the highest enrichment score in Figure 4, but also pathways such as “prostate cancer”, “glioma”, “tuberculosis”, etc. showed significantly elevated enrichment scores. Furthermore, Figure 5 shows significant enrichment in the C-type lectin receptor signaling pathway, being associated with elevated viability of the treated cells. These results could be linked to the significantly elevated levels of key players being involved in the MAPK signaling, as they do occur in all of these pathways, comparing them at the KEGG database.

Since targeted therapies have already been successfully used in several cancers to counteract the enormous survival advantage of cancer cells that establish a supportive TME [30], based on our results, the MEK inhibitor trametinib could be a potent therapeutical option for some MPM patients and would be interesting to be addressed. Further studies are underway and will confirm whether or not cell lines and “ex-vivo” culture of primary MPM treated with MEK inhibitors show response to treatment.

Fibroblasts pass different states of activation in a so called “life cycle” [19]. The normal, “quiescent” fibroblasts have widely been shown to neither underwent cell cycle progression nor secretion of activating soluble factors to the extracellular space and thereby would not influence the signaling pathway activity shown via the phosphorylation of specific recognition sequences. Independent of the origin of the fibroblast strain, the measured, therapeutically relevant activation of ERK and MEK remains promising and shows parallels to the situation and detected expression pattern in patient samples and previous cell culture models.

In addition, MEK inhibition by trametinib is thought to reprogram CD8+ T lymphocytes into memory stem cells, further enhancing their antitumor properties [33]. Through this reprogramming of T-lymphocytes, MEK inhibitors might act synergistically with established cisplatin treatment. As Kong et al. already demonstrated in other thoracic malignancies, the addition of MEK-inhibition reduces cisplatin resistance in squamous cell carcinoma of the lung [34]. Furthermore, ERK inhibition in combination with cisplatin (34) might prove to be a promising therapeutic option, as inhibition of these kinases counteracts the interaction between CAFs and tumor cells, thereby enhancing the apoptotic effect of cisplatin [35].

## 3. Materials and Methods

### 3.1. Study Design

We simulated paracrine signaling in vitro by treating MPM cell lines with conditioned medium (CM) from fibroblasts (FB) and vice versa. NCI-H2052 (epithelioid; lot-number: CRL-5915, ATCC, Manassas, VA, USA), MSTO-211H (biphasic; lot-number: CRL-2081, ATCC), and NCI-H2452 (sarcomatoid; lot-number: CRL-5946, ATCC) cell lines represented MPM. Embryonal myofibroblast cell lines, IMR-90 and MRC-5, were selected since they present a CAFs-like phenotype [36,37]. In addition, differences in proliferation rates, migratory behavior, apoptosis, necrosis, and viability were measured and used as a covariate for data analysis.

Kinase activity of treated samples, as well as control cells (conditioned medium from the cell line itself), were prepared by creating a total of 17 experimental conditions (5 Control + 2 of each MPM + 3 of each FB) and subsequently analyzed using the PamStation12 (PamGene, PamGene International B. V., HH ’s-Hertogenbosch, The Netherlands).

### 3.2. Cell Culture, Maintenance, Conditioned Medium Treatment, and Cell State Analysis

In short, all cells were cultivated in Rosswell Park Memorial Institute (RPMI)-1640 medium supplemented with 10% fetal calf serum and 1% penicillin/streptomycin (RPMI-mixture). Washing steps were performed twice in 5 mL of Dulbecco’s phosphate-buffered saline (DPBS) before media replacement. Trypsinization was performed with 2 mL of a 10% Trypsin-EDTA solution applied to the flask for 10 min at room temperature. CM was generated by applying two million cells of the desired cell type into T-175 flasks and a total volume of 24 mL of the RPMI-mixture mentioned above. After an incubation time of 48 h, the conditioned media was extracted and immediately used to treat awaiting cells. Treatment lasted for 24 h, after which cells were counted, thereby delivering proliferation rates. The ApoTox-Glo Triplex Assay kit (Promega Corporation, Fitchburg, WS, USA) was used to measure viability, necrosis, and apoptosis. The evaluation was carried out using the Promega GloMax Multi + Detection System. For assessment of MPM cell-invasive behavior under the influence of FB CM, a 24-Well Cell migration and Invasion Assay (Cell Biolabs, CA, USA) was used for all available MPM samples.

All samples that did not serve as positive controls went through treatment in technical triplicates in all analyses mentioned above. The experiment has been set up independently twice, resulting in biological replicates. All technical triplicates were summarized as a mean for all further calculations. Biological replicates were treated as separate samples within the data analysis.

### 3.3. Protein Extraction of Cells after Conditioned Medium Treatment

Following treatment, protein isolation was performed according to the protocol 1160 from the PamGene platform. Cells were lysed using M-PER Mammalian Protein Extraction Reagent containing HALT phosphatase inhibitor cocktail and HALTprotease inhibitor cocktail EDTA-free (Thermo Fisher Scientific, Waltham, MA, USA). Lysed cells were harvested by using a cell scraper. Lysates were stored in 5–20 μL aliquots at −80 °C. The protein concentration was determined via fluorometric quantification (Qubit, Thermo Fisher Scientific, Waltham, MA, USA) using the protein assay kit according to the manufacturers’ instructions.

### 3.4. Protein Tyrosine and Serine/Threonine Kinase Assay

Subsequently, also following manufacturers’ instructions, the Protein Tyrosine Kinase—(PTK Assay, PamGene) and the Serine/Threonine Kinase Assay (STK Assay, PamGene) were performed in order to determine changes in kinase activity compared to generated controls. Blocking the PamChip^®^-4 was by application of 30 µL of 2% BSA (PamGene) while preparing the master mix with the appertaining reagent kit for PTK or STK PamChip arrays (PamGene). 5 µg (PTK assay) or 0.5 µg (STK assay) of desired sample protein lysate were then applied.

### 3.5. Data Analysis

Image analysis of both PTK and STK assay was performed using the Bionavigator software (PamGene). Determination of kinetics for each phosphosite, normalization between spots, and cutoff calculation for countable phosphorylation of individual phosphosites have been performed according to the manufacturers’ instructions.

Statistical and graphical analyses of specific phosphosite phosphorylation levels were carried out using the R statistical programming environment V 4.0.2 (The R Project, Vienna, Austria). The Shapiro–Wilks test was applied to test for the normal distribution of each data set for ordinal and metric variables before exploratory data analysis. The resulting dichotomous variables underwent either the Wilcoxon–Mann–Whitney rank-sum test (nonparametric) or two-sided student’s *t*-test (parametric). To compare ordinal variables and factors with more than two groups, either the Kruskal–Wallis test (nonparametric) or ANOVA (parametric) was used to detect group differences.

Double dichotomous contingency tables were analyzed using Fisher’s exact test. To test the dependency of ranked parameters with more than two groups, Pearson’s chi-squared test was used. Correlations between metrics were tested applying Spearman’s rank correlation test and Pearson’s product-moment correlation testing for linearity.

Basic quality control of run data was performed by mean-vs.-variances plotting to find outliers in the target or sample level. True differences were calculated by correlation matrices analysis. Pathway analysis is based on the KEGG database and was performed using the “pathview” package of R. Differences were specified by −log_2_ fold changes between means (if parametric) or medians (if nonparametric) of compared groups. Significant pathway associations were identified by gene set enrichment analysis using the WEB-based GEne SeT AnaLysis Toolkit (WebGestalt) (48, 49). Each run was executed with 1000 permutations. Finally, all associations were ranked according to the false discovery rate (*p* < 0.05).

Due to the multiple statistical tests, the *p*-values were adjusted using the false discovery rate (FDR). The level of statistical significance was defined as *p* ≤ 0.05 after adjustment.

## 4. Conclusions

The factors secreted by fibroblasts into fibroblast CM significantly influence the phosphorylation of kinases, mainly affecting the MAPK signaling cascade in analyzed MPM cell lines. This pathway plays a decisive role in cancer progression since it affects cell proliferation, differentiation, migration, senescence, and apoptosis. Based on our results, MEK- or ERK- inhibitors could be potent agents for therapy. Additionally, the inhibitors might offer a positive synergistic effect in combination with cisplatin-based treatment, as the cancer-progressing factors would be inhibited and additionally cisplatin would enhance cells undergoing apoptosis. The synergistic effect hopefully enhances the therapeutic outcome and therefore the survival time of MPM patients.

## Data Availability

The data used to support the findings of this study are available from the corresponding author upon request.

## Supplementary Materials

The following supporting information can be downloaded at: https://www.mdpi.com/article/10.3390/ijms23063278/s1.
