# Supplementary material for: Cancer-Associated Fibroblasts Regulate Kinase Activity in Mesothelioma Cell Lines via Paracrine Signaling and Thereby Dictate Cell Faith and Behavior
Source: Int J Mol Sci. 2022 Mar 18;23(6):3278. doi: 10.3390/ijms23063278 (PMC8949651; doi:10.3390/ijms23063278)
Supplement: Supplementary file 1 [file ijms-23-03278-s001.zip › ijms-1604536-supplementary.pdf]

Table S1. Differences in viability rates associated with phosphorylation activity.

Gene	p-Value (Spearman)	Rho	p-Value (LM)	R-Squared	adjusted R-Squared
**Diff. viability % by RON_1346_1358**	0.0056	0.83	0.0890	0.36	0.27
**Diff. viability % by MAPK3_198_210_C203S**	0.0096	0.80	0.0330	0.50	0.43
**Diff. viability % by CD3Z_77_89**	0.0170	0.76	0.0890	0.36	0.27
**Diff. viability % by EGFR_1165_1177**	0.0260	0.73	0.1800	0.24	0.13
**Diff. viability % by GSK3B_210_222_C218S**	0.0260	0.73	0.0990	0.34	0.25
**Diff. viability % by JAK1_1027_1039**	0.0450	0.68	0.2900	0.16	0.04

Table S2. Differences in apoptotic rates associated with phosphorylation activity.

Gene	p-Value (Spearman)	Rho	p-Value (LM)	R-Squared	adjusted R-Squared
**Diff. Apoptose % by RB_804_816**	0.0001	0.96	0.0003	0.86	0.84
**Diff. Apoptose % by ARAF_297_307**	0.0005	0.92	0.0052	0.70	0.65
**Diff. Apoptose % by AKT1_320_332**	0.0010	0.90	0.0021	0.76	0.73
**Diff. Apoptose % by FAK2_572_584**	0.0017	0.88	0.0180	0.58	0.52
**Diff. Apoptose % by K2C6B_53_65**	0.0017	0.88	0.0012	0.80	0.77
**Diff. Apoptose % by PGFRB_572_584**	0.0017	0.88	0.0200	0.56	0.50
**Diff. Apoptose % by PTN11_580_590**	0.0017	0.88	0.0029	0.74	0.70
**Diff. Apoptose % by BTLA_252_262**	0.0026	0.86	0.0020	0.77	0.73
**Diff. Apoptose % by CD3Z_117_129**	0.0026	0.86	0.0036	0.73	0.69
**Diff. Apoptose % by K2C8_425_437**	0.0036	0.85	0.0290	0.52	0.45
**Diff. Apoptose % by CD3E_193_205**	0.0039	0.85	0.0180	0.57	0.51
**Diff. Apoptose % by CD3Z_116_128**	0.0039	0.85	0.0270	0.53	0.46
**Diff. Apoptose % by CDK1_9_21**	0.0039	0.85	0.0049	0.70	0.66
**Diff. Apoptose % by PDPK1_2_14**	0.0039	0.85	0.0014	0.79	0.76
**Diff. Apoptose % by PGFRB_771_783**	0.0039	0.85	0.0052	0.70	0.65
**Diff. Apoptose % by PTN6_531_541**	0.0039	0.85	0.0051	0.70	0.65
**Diff. Apoptose % by RASA1_453_465**	0.0039	0.85	0.0053	0.69	0.65
**Diff. Apoptose % by DCX_67_79**	0.0056	0.83	0.0460	0.46	0.38
**Diff. Apoptose % by HAVR2_257_267**	0.0056	0.83	0.0071	0.67	0.62
**Diff. Apoptose % by MK10_216_228**	0.0056	0.83	0.0038	0.72	0.68
**Diff. Apoptose % by ZAP70_313_325**	0.0056	0.83	0.0046	0.71	0.66
**Diff. Apoptose % by CTNB1_79_91**	0.0076	0.81	0.0022	0.76	0.73
**Diff. Apoptose % by EPOR_419_431**	0.0076	0.81	0.0160	0.59	0.53
**Diff. Apoptose % by FER_707_719**	0.0076	0.81	0.0170	0.58	0.52
**Diff. Apoptose % by FGFR2_762_774**	0.0076	0.81	0.0090	0.65	0.60
**Diff. Apoptose % by IRS2_626_638**	0.0076	0.81	0.0130	0.61	0.55
**Diff. Apoptose % by MK08_181_191**	0.0076	0.81	0.0140	0.60	0.55
**Diff. Apoptose % by PAXI_24_36**	0.0076	0.81	0.0050	0.70	0.66
**Diff. Apoptose % by RAF1_332_344**	0.0076	0.81	0.0045	0.71	0.67

**Diff. Apoptose % by VGFR2_1168_1180**	0.0076	0.81	0.0700	0.39	0.31
**Diff. Apoptose % by ZAP70_485_497**	0.0076	0.81	0.0024	0.75	0.72
**Diff. Apoptose % by EPB42_241_253**	0.0076	-0.81	0.0210	0.56	0.50
**Diff. Apoptose % by STK6_283_295**	0.0076	-0.81	0.0180	0.57	0.51
**Diff. Apoptose % by CD28_185_197**	0.0100	0.80	0.0270	0.53	0.46
**Diff. Apoptose % by P85A_600_612**	0.0100	0.80	0.0140	0.60	0.55
**Diff. Apoptose % by PGFRB_1002_1014**	0.0100	0.80	0.0012	0.80	0.77
**Diff. Apoptose % by PLCG1_764_776**	0.0100	0.80	0.0027	0.74	0.71
**Diff. Apoptose % by PLCG2_1191_1203_C1200S**	0.0100	0.80	0.0080	0.66	0.61
**Diff. Apoptose % by CREB1_126_138**	0.0100	-0.80	0.0150	0.60	0.54

Table S3. Details of the gene set enrichment analysis regarding FB CM treatment.

**geneSet**	**enrichmentScore**	**normalizedEnrichmentScore**	**pValue**	**size**	**leadingEdgeNum**	**userId**
**Rap1 signaling pathway**	0.70	1.88	0.0001	26	10	AKT1;CALM1;CRK;EGFR;FLT1;KDR;MAP2K1;MAPK12;MAPK3;RAP1B
**Oocyte meiosis**	0.84	1.77	0.0001	10	6	CALM1;CAMK2G;MAP2K1;MAPK12;MAPK3;RPS6KA1
**Fc epsilon RI signaling pathway**	0.78	1.78	0.0001	14	7	AKT1;MAP2K1;MAPK10;MAPK12;MAPK3;MAPK8;SYK
**MAPK signaling pathway**	0.68	1.89	0.0027	33	18	AKT1;ARAF;CRK;EGFR;ERBB2;FGFR2;FLT1;KDR;MAP2K1;MAPK10;MAPK12;MAPK3;MAPK8;MAPKAPK3;NTRK2;RAP1B;RPS6KA1;TP53
**Neurotrophin signaling pathway**	0.69	1.81	0.0001	24	14	AKT1;CALM1;CAMK2G;CRK;GSK3B;MAP2K1;MAPK10;MAPK12;MAPK3;MAPK8;NTRK2;RAP1B;RPS6KA1;TP53
**Signaling pathways regulating pluripotency of stem cells**	0.77	1.78	0.0025	14	10	AKT1;CTNNB1;FGFR2;FGFR3;GSK3B;JAK1;JAK2;MAP2K1; MAPK12;MAPK3
**Axon guidance**	0.70	1.72	0.0047	16	3	CAMK2G;GSK3B;MAPK3

**Fc gamma R-mediated phagocytosis**	0.71	1.65	0.0128	15	7	AKT1;CRK;MAP2K1;MAPK3;MARCKS;NCF1;SYK
**Long-term potentiation**	0.74	1.64	0.0093	12	6	CALM1;CAMK2G;MAP2K1;MAPK3;RAP1B;RPS6KA1
**Melanogenesis**	0.76	1.63	0.0319	10	5	CALM1;CAMK2G;GSK3B;MAP2K1;MAPK3
**JAK-STAT signaling pathway**	-0.27	-0.58	0.9632	14	1	MTOR
**Th17 cell differentiation**	-0.33	-0.73	0.8557	16	2	MTOR;NFKB1
**AMPK signaling pathway**	-0.58	-1.22	0.2247	12	3	CFTR;LIPE;MTOR
**Acute myeloid leukemia**	-0.55	-1.15	0.2995	11	4	BAD;CSF1R;MTOR;NFKB1
**Viral carcinogenesis**	-0.40	-0.91	0.5990	18	3	BAD;NFKB1;REL
**Insulin signaling pathway**	-0.36	-0.88	0.6531	23	6	BAD;LIPE;MTOR;PHKA1;PRKAR2A;RPS6
**Autophagy**	-0.41	-0.86	0.6667	12	2	BAD;MTOR
**MicroRNAs in cancer**	-0.35	-0.80	0.7384	19	3	CDC25A;MTOR;NFKB1

Table S4. Details of gene set enrichment analysis regarding apoptosis of cells.

**geneSet**	**enrichmentScore**	**normalizedEnrichmentScore**	**pValue**	**size**	**leadingEdgeNum**	**userId**
**MAPK signaling pathway**	0.55	1.96	0.0031	24	13	ARAF;ERBB2;FGFR1;FGFR2;MAPK10;MAPK12;MAPK3;MAPK8;MAPKAPK3;NTRK2;PDGFRB;RAF1;RASA1

**Rap1 signaling pathway**	0.53	1.75	0.0159	19	8	CTNNB1;FGFR1;FGFR2;LAT;MAPK12;MAPK3;PDGFRB;RAF1
**Prostate cancer**	0.58	1.88	0.0001	18	12	ARAF;CTNNB1;ERBB2;FGFR1;FGFR2;GSK3B;MAPK3;MTOR;PDGFRB;PDPK1;RAF1;RB1
**Glioma**	0.59	1.79	0.0088	15	8	ARAF;CAMK2G;CDK4;MAPK3;MTOR;PDGFRB;RAF1;RB1
**Tuberculosis**	0.66	1.82	0.0045	11	7	CAMK2G;MAPK10;MAPK12;MAPK3;MAPK8;RAF1;SYK
**Pancreatic cancer**	0.57	1.76	0.0196	14	9	ARAF;CDK4;ERBB2;MAPK10;MAPK3;MAPK8;MTOR;RAF1;RB1
**Choline metabolism in cancer**	0.61	1.65	0.0366	11	7	MAPK10;MAPK3;MAPK8;MTOR;PDGFRB;PDPK1;RAF1
**Kaposi sarcoma-associated herpesvirus infection**	0.49	1.64	0.0323	21	10	CDK4;CTNNB1;MAPK10;MAPK12;MAPK3;MAPK8;MTOR;RAF1;RB1;SYK
**ErbB signaling pathway**	0.51	1.66	0.0227	19	12	ARAF;CAMK2G;CBL;ERBB2;ERBB4;GSK3B;MAPK10;MAPK3;MAPK8;MTOR;PTK2;RAF1
**Fc epsilon RI signaling pathway**	0.59	1.66	0.0175	13	8	LAT;MAPK10;MAPK12;MAPK3;MAPK8;PDPK1;RAF1;SYK
**Calcium signaling pathway**	-0.55	-1.42	0.1467	13	3	CHRM5;PHKA1;RYR1
**AMPK signaling pathway**	-0.33	-0.79	0.6861	10	3	FOXO3;LIPE;PFKFB3
**Insulin signaling pathway**	-0.41	-1.12	0.3774	19	3	LIPE;PHKA1;PRKAR2B
**Prolactin signali**	-0.34	-0.83	0.6250	11	2	ESR1;FOXO3

**ng pathway**						

Table S5. Details of gene set enrichment analysis regarding viability of cells.

**geneSet**	**enrichmentScore**	**normalizedEnrichmentScore**	**pValue**	**size**	**leadingEdgeNum**	**userId**
**AMPK signaling pathway**	-0.38	-1.10	0.3364	11	1	FOXO3
**C-type lectin receptor signaling pathway**	0.72	1.66	0.0051	12	6	AKT1;CALM1;MAPK12;MAPK3;NFKB1;SYK
**Acute myeloid leukemia**	0.54	1.24	0.1802	10	4	AKT1;MAP2K1;MAPK3;NFKB1
**Oocyte meiosis**	0.60	1.36	0.1047	10	6	CALM1;CAMK2G;MAP2K1;MAPK12;MAPK3;RPS6KA1
**Central carbon metabolism in cancer**	0.49	1.20	0.2309	15	9	AKT1;ERBB2;FGFR2;FGFR3;MAP2K1;MAPK3;MET;RAF1;TP53
**Phospholipase D signaling pathway**	0.51	1.21	0.2250	13	4	AKT1;MAP2K1;MAPK3;SYK
**Toxoplasmosis**	0.54	1.25	0.2010	11	4	AKT1;MAPK12;MAPK3;NFKB1
**MicroRNAs in cancer**	0.48	1.21	0.2228	18	11	CDC25A;CRK;ERBB2;FGFR3;IRS1;MAP2K1;MARCKS;MET;NFKB1;RAF1;TP53
**Ras signaling pathway**	0.44	1.25	0.1673	32	10	AKT1;CALM1;FGFR2;FLT1;KDR;MAP2K1;MAPK3;NFKB1;RAP1B;REL
**Estrogen signaling pathway**	0.54	1.23	0.1875	10	5	AKT1;CALM1;CREB1;MAP2K1;MAPK3
**Human papillomavirus infection**	0.45	1.21	0.2096	23	10	AKT1;CREB1;CTNNB1;GSK3B;JAK1;MAP2K1;MAPK3;NFKB1;RBL2;TP53
**Non-small cell lung cancer**	-0.34	-1.13	0.3182	19	4	ARAF;FOXO3;PRKCB;RB1
